# Obesity pharmacotherapy reimagined: The era of multi-receptor agonists and next-generation metabolic modulators, perspectives and controversies

**DOI:** 10.1016/j.metop.2026.100463

**Published:** 2026-03-23

**Authors:** Ioannis G. Lempesis, Maria Dalamaga

**Affiliations:** a Division of Sleep Medicine, Harvard Medical School, Boston, MA, USA; b Medical Chronobiology Program, Division of Sleep and Circadian Disorders, Departments of Medicine and Neurology, Brigham and Women's Hospital, Boston, MA, USA; c Department of Biologic Chemistry, School of Medicine, National and Kapodistrian University of Athens, Mikras Asias 75, 115 27, Athens, Greece

**Keywords:** Amycretin, Amylin, Amylin analogs, Bimagrumab, Cagrilintide, GLP-1 receptor agonists, Lean mass, Metabolic-associated steatotic liver disease, Multi-receptor agonists, Obesity, Obesity pharmacotherapy, Orforglipron, Oral GLP-1 agonists, Retatrutide, Survodutide, Weight loss

## Abstract

Obesity affects over 2 billion adults globally, with projections indicating that nearly two-thirds of adults will be affected by 2050. Glucagon-like peptide-1 receptor agonists (GLP-1RAs) have transformed obesity treatment, achieving weight loss previously considered attainable only with bariatric surgery. However, GLP-1-based therapies have revealed important limitations, including weight loss plateaus, substantial inter-individual variability, and weight regain upon discontinuation, underscoring the need for next-generation approaches. Existing reviews have focused predominantly on approved GLP-1RAs, with limited synthesis of emerging multi-receptor agonists, oral formulations, and body composition–targeted agents, while guidance on treatment personalization and sequencing strategies remains limited.

This review examines the evolving landscape of obesity pharmacotherapy beyond injectable GLP-1RAs. Oral GLP-1 agonists, including orforglipron, offer comparable efficacy to injectables while potentially improving global accessibility by eliminating cold-chain requirements and simplifying manufacturing. Multi-receptor agonists represent the most transformative developments: triple agonists such as retatrutide achieve weight reductions of 20–24%, while dual GLP-1/glucagon agonists like survodutide and mazdutide show strong efficacy with particular promise for metabolic-associated steatotic liver disease. Maridebart cafraglutide, combining GLP-1 agonism with glucose-dependent insulinotropic polypeptide (GIP) antagonism, enables once-monthly dosing. The amylin pathway has re-emerged through long-acting analogs (cagrilintide, eloralintide) and unimolecular co-agonists (amycretin), achieving weight reductions up to 24% via distinct neuroendocrine circuits. Body composition optimization through agents like bimagrumab addresses lean mass preservation during potent anorectic therapy. Personalized approaches, including setmelanotide for monogenic obesity, exemplify precision pharmacotherapy.

Collectively, these advances signal a shift from appetite-centric weight loss toward integrated metabolic, neuroendocrine, and body-composition–focused disease modification. The next epoch of obesity pharmacotherapy will be defined by multi-receptor strategic combinations, targeted approaches to preserve lean mass, and personalized treatment algorithms. Critical priorities include phenotype-stratified trials, long-term safety surveillance, pediatric obesity research, and implementation science to ensure equitable global access. Balancing pharmacologic innovation with sustainable, equitable implementation remains the defining challenge ahead.

## List of abbreviations

AMY1R: amylin receptor subtype 1
ASCVD: atherosclerotic cardiovascular disease
BBS: Bardet–Biedl syndrome
BMI: body mass index
BP: blood pressure
CKD: chronic kidney disease
CNS: central nervous system
CV: cardiovascular
GI: gastrointestinal
GIP: glucose-dependent insulinotropic polypeptide
GLP-1: glucagon-like peptide-1
GLP-1RA: glucagon-like peptide-1 receptor agonist
HbA1c: glycated hemoglobin
HR: heart rate
IV: intravenous
MASLD: metabolic-associated steatotic liver disease
MASH: metabolic-associated steatohepatitis
MC4R: melanocortin-4 receptor
PCSK1: proprotein convertase subtilisin/kexin type 1
POMC: proopiomelanocortin
RA: receptor agonist
SC: subcutaneous
T2DM: type 2 diabetes mellitus
WHO: World Health Organization

## 1 Introduction: From GLP-1 success to the next therapeutic question

Obesity has increased substantially over the past few decades, emerging as a major global public health challenge that significantly increases the risk and worsens the prognosis of several diseases, including cardiometabolic, communicable, and malignant diseases, making its treatment of pivotal importance [[1], [2], [3], [4], [5], [6], [7], [8], [9], [10], [11]]. According to the Global Burden of Disease Study 2021, an estimated 2.11 billion adults had overweight or obesity in 2021, with projections indicating that nearly two-thirds of adults over age 25 will be affected by 2050 and approximately one-third will have obesity specifically [12]. Despite this burden, obesity pharmacotherapies remain underprescribed. Although over 50% of adults meet eligibility criteria for pharmacotherapy, only a small minority currently receive these agents, with prescribing patterns standing in contrast to those for type 2 diabetes mellitus (T2DM) and hypertension [13].

The remarkable success of glucagon-like peptide-1 (GLP-1) receptor agonists (GLP-1RAs) has transformed the therapeutic landscape for obesity, achieving levels of weight loss previously considered attainable only with bariatric surgery [2,[14], [15], [16], [17], [18], [19], [20], [21], [22]]. Semaglutide and tirzepatide demonstrate that obesity can be effectively managed by targeting gut-brain hormonal pathways, reshaping not only clinical expectations but also societal perceptions [2,[23], [24], [25], [26]]. For context, bariatric surgery achieves 5-year total weight loss of 20–25% with Roux-en-Y gastric bypass and 16–19% with sleeve gastrectomy, representing the historical benchmark for durable weight reduction [27,28]. A recent network meta-analysis demonstrated that although metabolic/bariatric surgery remains superior overall, with approximately 10% greater total weight loss than GLP-1RAs, tirzepatide approaches surgical efficacy without a statistically significant difference compared with bariatric procedures [29,30]. Real-world data suggest that bariatric surgery achieves approximately 28% total weight loss compared with approximately 10% with GLP-1RAs; however, this gap narrows substantially with newer dual and triple agonists [31]. These agents have fundamentally redefined obesity as a chronic, pharmacologically modifiable disease requiring long-term medical management, analogous to other cardiometabolic conditions such as hypertension and T2DM [32].

Despite their significance, GLP-1-based therapies have also revealed important limitations that define the next therapeutic frontier [22,33,34]. Weight loss typically plateaus at approximately 18 months, with real-world efficacy being often lower than trial results (approximately 8% in patients with T2DM and 11% in patients without T2DM at 60 weeks with semaglutide) [35]. Furthermore, weight regain is common upon discontinuation, with up to two-thirds of lost weight regained within one year, even when lifestyle interventions are maintained [36]. Substantial inter-individual variability exists, with some patients experiencing minimal weight loss while others achieve reductions exceeding 30% [35]. These observations underscore critical gaps in our understanding of optimal treatment strategies, the mechanisms underlying the superior efficacy of multi-receptor approaches, long-term safety profiles, and methods to maximize individual response while minimizing weight recurrence.

As therapeutic and societal demand grows and mechanistic understanding advances, the field is shifting rapidly toward multi-agonist, combination, and next-generation therapies designed to overcome the limitations of single-hormone treatment [2,37,38]. Multi-receptor agonists combining GLP-1 with glucose-dependent insulinotropic polypeptide (GIP), glucagon, or amylin demonstrate enhanced weight loss and metabolic benefits through synergistic mechanisms that augment energy expenditure, lipolysis, and satiety beyond GLP-1 alone [39].

Despite the rapid expansion of the obesity pharmacotherapy armamentarium, critical gaps persist in the current literature. Existing reviews and meta-analyses have focused predominantly on approved GLP-1RAs, with limited synthesis of the mechanistic diversity and comparative positioning of emerging multi-receptor agonists, oral formulations, and anabolic-supportive agents. Furthermore, guidance on the personalization of pharmacological interventions based on patient phenotypes and comorbidities remains limited, and strategies for sequencing, combining, and transitioning between agents have not been comprehensively addressed. Finally, the implications of next-generation pharmacotherapies for long-term body composition preservation, functional outcomes, and global accessibility require systematic evaluation.

**Fig. 1:**
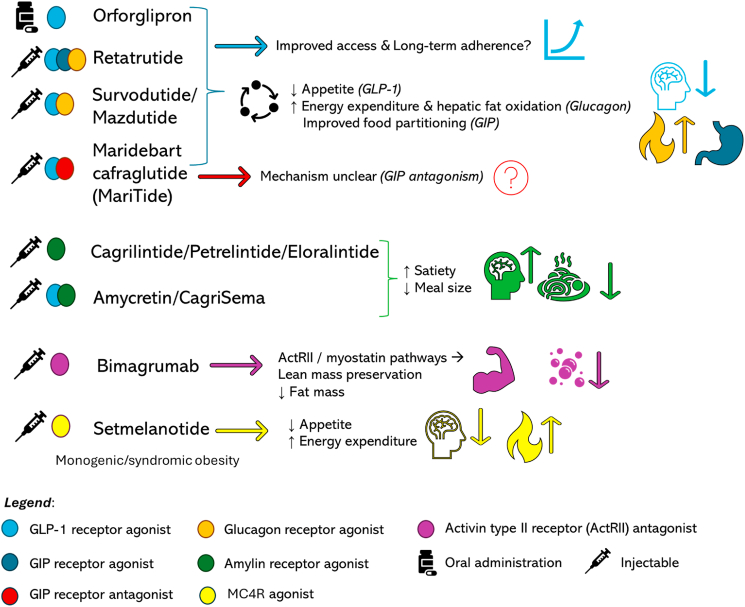
Emerging anti-obesity pharmacotherapies expand beyond glucagon-like peptide-1 (GLP-1) receptor agonists (RAs). These include oral GLP-1, multi-receptor strategies (GLP-1, glucose-dependent insulinotropic polypeptide (GIP), glucagon), amylin analogs, GLP-1 and amylin receptor agonist combinations, and non-incretin approaches targeting appetite, energy expenditure, and body composition. MC4R, melanocortin-4 receptor.

This review aims to bridge these gaps by providing a comprehensive, mechanistically organized synthesis of the evolving obesity pharmacotherapy landscape beyond injectable GLP-1RAs. We examine the therapeutic potential of oral GLP-1RAs, dual and triple multi-receptor agonists, amylin-based therapies, and body composition–targeted agents, while critically appraising the evidence regarding efficacy ceilings, safety trade-offs, and long-term maintenance strategies. Additionally, we discuss principles of personalized treatment selection, implementation challenges, and the controversies likely to shape the integration of these agents into clinical practice. A summary of the principal therapies currently under development is illustrated in Fig. 1. An overview of emerging pharmacological strategies beyond GLP-1 receptor agonism, including their mechanistic targets, efficacy profiles, and potential clinical positioning, is summarized in Table 1.

**Table 1:** Emerging obesity pharmacotherapies beyond GLP-1 receptor agonism.

Agent/Class	Primary receptor targets	Mechanism	Route/dosing	Mean weight loss in trials	Key advantages	Key limitations/concerns	Potential ideal patient profile
Orforglipron (oral GLP-1RA) [40,41]	GLP-1	Appetite suppression, delayed gastric emptying	Oral, once daily	Up to ∼9–10%, 36 mg at 72 weeks (phase 3, patients with T2DM and BMI ≥27 kg/m^2^); higher in phase 2 up to 14.7%, 45 mg at 36 weeks	Oral administration, no cold chain, scalable manufacturing	Long-term adherence unknown; GI effects	Patients reluctant to injections; primary care settings; global access contexts
Semaglutide (injectable GLP-1RA) [42]	GLP-1	Appetite suppression, improved glycemic control	SC, once weekly	Mean ∼ 10.2% at 208 weeks (2.4 mg, SELECT trial)	Proven CV benefit; robust long-term data	Lean mass loss; weight regain after discontinuation	Obesity with T2D, ASCVD, CKD
Tirzepatide (dual agonist) [30]	GLP-1/GIP	Enhanced satiety, nutrient partitioning	SC, once weekly	∼20.9% at 36 weeks, ∼25.3% at 88 weeks (SURMOUNT-4)	Superior efficacy vs GLP-1 alone; cardiometabolic benefit	Lean mass loss; long-term safety still evolving	Severe obesity with metabolic complications
Retatrutide (triple agonist) [43]	GLP-1/GIP/Glucagon	Satiety, ↑ energy expenditure, improved body composition	SC, once weekly	∼20–24% (phase 2), up to 24.2% at 48 weeks	Increased efficacy; improved metabolic/liver outcomes	Physiological limits of weight loss; HR and hepatic concerns; long-term safety unknown	Severe obesity; potential future bariatric alternative; metabolic syndrome, MASLD
Survodutide (dual agonist) [44,45]	GLP-1/Glucagon	Appetite suppression; lipid oxidation; visceral fat reduction	SC, once weekly	Up to ∼15%, 46 weeks, depending on dose and estimand;	Strong liver fat reduction (at least 30% 48 weeks); MASH indication; Visceral fat loss	GI side effects; Glucagon-related CV/hepatic safety; limited long-term data	Obesity with MASLD/MASH
Mazdutide (dual agonist) [46,47]	GLP-1/Glucagon	Appetite suppression, ↑ energy expenditure, hepatic lipid oxidation, ↓ triglycerides, ↓ serum uric acid	SC, once weekly	11–14 % at 48 weeks (at doses of 4–6 mg); up to 21% at 20 weeks (at higher doses of up to 16 mg) Phase 1	Potential for MASH, metabolic improvement	GI side effects, limited data	Obesity with metabolic dysfunction
Maridebart cafraglutide (MariTide) [48]	GLP-1, GIP (antagonist)	Appetite suppression, nutrient partitioning	SC, monthly	Up to 16.2% at 52 weeks in adults with obesity without T2DM, and up to 19.9% with obesity and T2DM, with improvements in glycemic control (↓ HbA1c up to 1.6 %).	Monthly dosing, ↑ half-life of approximately 21 days, ↑ weight loss	Mechanism unclear; GI adverse effects; limited long-term data	Obesity needing less frequent dosing
Cagrilintide (amylin analog) [49]	amylin	Meal-size control, satiation	SC, once weekly	Up to ∼10.8% at 26 weeks, (phase 2)	Distinct CNS pathway; complements incretins	GI tolerability; limited monotherapy data	GLP-1 intolerance; combination regimens
CagriSema (combo) [50,51]	GLP-1/Amylin	Dual satiety pathways; delayed gastric emptying, improved body composition	SC, once weekly	Up to 20.4% (without T2DM) and up to 13.7% with T2DM at 68 weeks	↑ efficacy with mechanistic diversity; enhanced satiety; lean mass preservation	Complexity of combination therapy; GI side effects, titration needed	Obesity with T2DM, need for lean mass preservation
Eloralintide (amylin RA) [52]	Selective AMY1R	Potent satiation; improved body composition	SC, once weekly	Up to ∼20% at 48 weeks	↑ efficacy; distinct receptor selectivity; monotherapy or add-on	GI side effects; fatigue; long-term safety unknown	Incretin-intolerant patients
Amycretin (unimolecular co-agonist) [53]	GLP-1/Amylin	Integrated satiety signaling	SC, once weekly	Up to ∼24% at 36 weeks	Single-molecule convenience; no plateau observed	Early-phase data only	Future high-efficacy maintenance strategies
Bimagrumab (body composition agent) [54]	Activin type II receptor (antagonist)	Fat loss with lean mass gain	IV/SC (trial-dependent)	Up to 6.5% weight loss; 20.5% fat mass reduction; 3.6% increase in lean mass at 48 weeks	Preserves or ↑ muscle mass	Limited weight loss alone; early phase, IV route	Sarcopenic obesity; combination therapy
Setmelanotide [55]	MC4R	Appetite suppression, ↑ energy expenditure	SC, daily	10–25% at one year (monogenic obesity)	Targeted for rare genetic obesity; rapid and sustained weight loss; improvement in hunger-related behaviors; favorable safety profile	Limited to syndromic/monogenic cases; injection site reactions; skin hyperpigmentation; nausea, vomiting, and diarrhea; rare neuropsychiatric effects and sexual arousal	Monogenic/syndromic obesity (e.g. BBS, POMC)

**List of abbreviations:** AMY1R, amylin receptor subtype 1; ASCVD, atherosclerotic cardiovascular disease; BBS, Bardet–Biedl syndrome; CKD, chronic kidney disease; CNS, central nervous system; CV, cardiovascular; GI, gastrointestinal; GIP, glucose-dependent insulinotropic polypeptide; GLP-1, glucagon-like peptide-1; HbA1c, glycated hemoglobin; HR, heart rate; IV, intravenous; MASLD, metabolic-associated steatotic liver disease; MASH, metabolic-associated steatohepatitis; MC4R, melanocortin-4 receptor; PCSK1, proprotein convertase subtilisin/kexin type 1; POMC, proopiomelanocortin; RA, receptor agonist; SC, subcutaneous; T2DM, type 2 diabetes mellitus.

## 2 Expanding the incretin paradigm: oral GLP-1 agonists

Orforglipron, the first effective non-peptide oral GLP-1RA, marks a significant step toward reshaping obesity care. Early trials showed meaningful weight loss and glycemic benefits, positioning orforglipron as a potential first-line option for individuals hesitant about injectable therapies [56]. In the phase 3 ATTAIN-2 trial, orforglipron achieved mean bodyweight reductions of up to approximately 9.6% at 72 weeks in adults with obesity and T2DM, with 45.6% of participants achieving at least 10% weight loss at the highest dose (36 mg), alongside glycated hemoglobin (HbA1c) reductions of up to 1.66% [40]. Phase 2 data showed even greater efficacy, with mean reductions in body weight of up to 12.6% at 26 weeks and up to 14.7% at 36 weeks, depending on the dose [57], with 46-75% of participants achieving ≥10% weight loss at 36 weeks [41]. These outcomes position orforglipron as comparable to injectable GLP-1RAs while offering the convenience of oral administration without food or water restrictions, enabled by its 79.1% bioavailability as a small-molecule agent [40]. The significance of orforglipron as the first effective non-peptide oral GLP-1RA for obesity care lies in its pharmacologic and practical advantages over peptide-based agents, including oral semaglutide. Unlike oral semaglutide, which requires strict fasting and water intake protocols due to its peptide nature and absorption enhancer, orforglipron is a small molecule that can be taken without meal-timing restrictions and demonstrates robust, dose-dependent weight loss efficacy. This represents a meaningful advance in obesity pharmacotherapy, potentially improving patient adherence and expanding access to GLP-1RA therapy for those who prefer oral medications or cannot tolerate injections [41]**.**

Beyond the convenience of oral administration, oral incretin therapies may improve global accessibility by removing cold-chain requirements associated with injectable therapies, and lowering manufacturing complexity. Small-molecule GLP-1RA medicines are expected to be less expensive to manufacture, require no cold storage, and in most cases can be taken without regard to timing of meal ingestion, which may improve adherence while expanding global access [58]. Notably, the World Health Organization (WHO) has emphasized that emerging oral formulations may further improve production, distribution, and access by simplifying logistics and storage, particularly in regions where cold-chain infrastructure poses barriers to treatment delivery [59]. Importantly, current production capacity for GLP-1RA therapies could cover less than 10% of people living with obesity globally, even under the highest projected scenarios, underscoring the urgent need for scalable manufacturing solutions [59].

Patient preferences and adherence patterns further support the potential impact of oral formulations. Initial patient preferences strongly favor once-daily oral over once-weekly injectable therapies (76.5% vs 23.5%), though this gap narrows when specific administration procedures are considered [60]. Real-world evidence has shown that while injectable and oral semaglutide achieve similar glycemic control and weight loss outcomes, persistence on therapy is longer with injectable formulations, suggesting that factors beyond route of administration, including dosing complexity, gastrointestinal (GI) tolerability, and individual patient characteristics, influence long-term treatment success [61]. Oral semaglutide requires strict administration 30 min before food or other medications with limited water, which may limit effective use, whereas the lack of dietary restrictions of orforglipron may offer practical advantages for sustained adherence [40].

As obesity prevalence continues to rise worldwide, oral agents could meaningfully expand treatment reach. However, realizing this potential requires addressing not only manufacturing scalability and cost but also health system preparedness, equitable distribution strategies, and integration into primary care platforms to ensure that oral GLP-1RA therapies reach populations most in need.

## 3 Multi-receptor agonism as metabolic reprogramming

The most transformative and anticipated agents currently in development are multi-receptor agonists, notably those that activate GLP-1, GIP, and glucagon receptors [34,37,[62], [63], [64], [65], [66]]. Among these, triple agonists such as retatrutide and dual agonists like survodutide and mazdutide, are at the forefront of clinical development. These agents leverage complementary mechanisms:GLP-1R activation drives satiety and appetite suppression, GIP receptor activation enhances insulin secretion and may improve nutrient partitioning, while glucagon receptor activation increases energy expenditure and promotes hepatic lipid oxidation [38].

Among these multi-agonists, retatrutide has received increasing interest [[43], [67], [68], [69]]. Its triple-agonist mechanism combines complementary effects:profound satiety (via GLP-1), improved food partitioning (via GIP), and increased energy expenditure caused by glucagon receptor activation [68]. Phase 2 results suggest remarkable weight loss trajectories, that approach or exceed 20-24% (8 and 12 mg) at 48 weeks, putting retatrutide on track to potentially replace the existing incretin-dominated standard of care [43]. Phase 3 trials (TRIUMPH program) are ongoing to evaluate retatrutide for obesity, obstructive sleep apnea, and knee osteoarthritis, with primary endpoints including percent change in body weight and disease-specific outcomes [70]. Overall, retatrutide represents one of the most effective investigational agents for weight loss and glycemic control in obesity and T2DM, but long-term cardiovascular safety and comparative studies with other agents are still needed [71].

Survodutide (BI-456906), a dual glucagon/GLP-1RA, has demonstrated exceptional efficacy in phase 2 trials [65,[44], [72], [73]]. By combining GLP-1–mediated appetite suppression with glucagon-driven energy expenditure and lipid oxidation increases, survodutide resulted in significant weight reductions (up to −14.9% at 46 weeks) [45], as well as compelling reductions in liver fat (at least 30% at 48 weeks) [44]. However, the addition of glucagon receptor activity introduces safety considerations, as excessive activation may increase heart rate, hepatic aminotransferases, and potentially exacerbate cardiovascular (CV) or hepatic risk in susceptible individuals [74]. The optimal balance of receptor activation to maximize efficacy while minimizing adverse effects remains an area of active investigation.

Mazdutide, another dual glucagon/GLP-1RA, has shown increased efficacy particularly in Asian populations. In the phase 3 GLORY-1 trial, mazdutide at 6 mg achieved mean weight reductions of 14.01% at 48 weeks, with 49.5% of participants achieving at least 15% weight loss [46]. Higher doses have demonstrated even greater potential. A phase 1 study of 16 mg mazdutide demonstrated weight loss of 20–21% at 20 weeks without reaching a plateau [47]. Beyond weight reduction, its glucagon agonism appears to confer unique metabolic benefits, including pronounced improvements in triglycerides, alanine aminotransferase, liver fat content, and notably serum uric acid levels. These effects are potentially attributable to glucagon-driven hepatic lipid oxidation and purine metabolism [46].

Maridebart cafraglutide (MariTide) represents a mechanistically distinct approach, combining GLP-1 receptor agonism with GIP receptor antagonism as a long-acting peptide-antibody conjugate [48]. In phase 2 trials, maridebart cafraglutide achieved weight reductions of up to 16.2% at 52 weeks in adults with obesity without T2DM (treatment policy estimand), and up to 19.9% based on the efficacy estimand, without reaching a weight plateau [48]. Its 21-day half-life enables once-monthly or less frequent dosing, potentially improving long-term adherence. Intriguingly, how both GIP receptor agonism (with tirzepatide) and antagonism (with maridebart cafraglutide) promote weight reduction when combined with GLP-1 agonism remains incompletely understood, representing a key mechanistic question for the field.

Multi-agonists are particularly promising for obesity-associated liver disease, including metabolic-associated steatotic liver disease (MASLD) and metabolic-associated steatohepatitis (MASH). Survodutide and similar agents have shown reductions in hepatic steatosis and improvements in liver histology, supporting their dual metabolic and hepatologic indications. [44]. The parallel development and potential use of survodutide in both obesity and MASH reflects a broader trend toward therapies with dual metabolic and hepatologic indications. As these therapies advance through late-phase trials, the field must define the physiological limits of weight reduction and clarify long-term safety, especially regarding glucagon-mediated effects on the liver and CV system [[75], [76], [77], [78], [79], [80], [81]].

## 4 Amylin pathway Re-emergence and combinations: revisiting a powerful satiety pathway

Amylin is an endogenous hormone with potent effects on meal-size regulation, gastric emptying, and hindbrain-mediated satiety [[82], [83], [84], [85], [86]]. Although earlier amylin analogs offered modest benefits, the development of long-acting molecules such as cagrilintide and petrelintide has revived interest in this pathway [[49], [50], [51], [87], [88], [89], [90]]. Amylin operates through neuroendocrine circuits distinct from those of GLP-1, acting primarily on the area postrema and nucleus of the solitary tract in the hindbrain to induce satiation, while also modulating food-reward pathways through hypothalamic and ventral tegmental area receptors [85]. Unlike GLP-1, which exerts its satiety effects through direct central nervous system action, the effects of amylin are mediated by humoral action at amylin and calcitonin receptors in the caudal hindbrain, providing a mechanistically complementary pathway for appetite suppression [85]**.**

Pharmacologic advances in long-acting amylin analogs have transformed the therapeutic potential of this pathway. Cagrilintide, a long-acting acylated amylin analog with high homology to native amylin, demonstrated dose-dependent weight reductions of 6.0–10.8% at 26 weeks in phase 2 trials, with the 4.5 mg dose achieving greater weight loss than liraglutide 3.0 mg (10.8% vs 9.0%) [49]**.** Importantly, weight-loss trajectories had not plateaued by week 26, suggesting potential for greater efficacy with extended treatment [91]. Eloralintide, a selective amylin RA with 12-fold greater potency at the human amylin receptor (AMY1R) compared with the calcitonin receptor, produced substantial and dose-dependent weight loss in adults with obesity or overweight without T2DM [52]. Over 48 weeks, eloralintide achieved up to approximately 20% mean bodyweight reduction, supporting its potential as an obesity therapy with a safety and tolerability profile comparable to incretin-based therapies [52].

The combination of cagrilintide with semaglutide (CagriSema) illustrates the power of rational pairing [50,51]. CagriSema consistently achieves weight reductions of approximately 20–22%, comparable to tirzepatide but through a mechanistically distinct pathway, which may offer advantages in tolerability or sequencing [90,51]. In phase 3 trials, CagriSema demonstrated mean weight reductions of 20.4% in adults without T2DM [[13], [51]].7% in those with T2DM at 68 weeks, alongside HbA1c reductions of up to ∼2% and improvements in continuous glucose-monitoring parameters including time in range [50]. The combination was generally well tolerated, with GI adverse events that were primarily transient and mild to moderate in severity [50,51].

Unimolecular co-agonists represent the next evolution in amylin-based pharmacotherapy. Amycretin, an unimolecular GLP-1 and amylin RA, administered once-weekly subcutaneously, produced marked and dose-dependent weight loss in adults with overweight or obesity in a phase 1b/2a randomized controlled trial [53]. Across doses up to 60 mg, amycretin achieved up to approximately 24% mean bodyweight reduction over 36 weeks, with a safety and tolerability profile consistent with established GLP-1 and amylin-based therapies, and no evidence of an early weight-loss plateau [53]. By combining bioactive domains of GLP-1 and amylin in a single molecule, amycretin offers a more convenient, patient-centered approach compared with fixed-dose combinations [92].

Overall, amylin analogs are uniquely positioned to complement incretin-based therapies, as their satiety actions rely on distinct neural circuits. This pathway will likely remain a key part of post–GLP-1RA innovation, whether as monotherapy for patients who cannot tolerate incretin-based therapies or in combination regimens to achieve weight loss approaching or exceeding that of bariatric surgery.

## 5 Beyond weight loss: Body composition and functional outcomes

Potent anorectic agents have reignited concern about the loss of lean mass during rapid weight reduction [[93], [94], [95]]. Lean mass loss typically comprises 25–40% of total weight loss with GLP-1RAs and dual agonists, with tirzepatide and semaglutide at higher doses being among the least effective in preserving lean mass despite achieving the greatest overall weight reduction [96]. While reductions in muscle volume appear largely proportional to total weight loss and may be accompanied by improvements in muscle quality through reduced intramuscular fat infiltration, the absolute loss of lean tissue raises concerns about long-term functional consequences, particularly in vulnerable populations [97].

This has opened the door for therapies specifically targeting muscle preservation [98]. The most notable is bimagrumab, an activin type II receptor antagonist that selectively reduces fat mass while increasing lean mass [99,100]. In phase 2 trials, bimagrumab produced a modest, up to 6.5% weight loss, 20.5% reduction in fat mass alongside a 3.6% increase in lean mass over 48 weeks, with accompanying improvements in glycemic control in individuals with T2DM and obesity [54]. Although bimagrumab alone does not achieve the magnitude of weight reduction seen with incretin therapies, its distinctive effects on body composition suggest potential in combination regimens [54,98]. The phase 2 BELIEVE trial demonstrated that bimagrumab combined with semaglutide achieved weight reduction of up to 22.1% at 72 weeks, with nearly up to 93% of weight loss attributable to fat loss [101]. Similarly, the COURAGE trial (interim 26 week analysis) showed that combining semaglutide with trevogrumab (anti-myostatin) preserved 50–51% of lean mass, while adding garetosmab (anti-activin A) preserved up to 80% of lean mass while further enhancing fat loss [[102], [103], [104]].

Future obesity care may involve pairing appetite-reducing agents with anabolic or anti-catabolic therapies to optimize metabolic outcomes and improve functional status, particularly in older adults or individuals with sarcopenic obesity [95]. Resistance training remains the cornerstone intervention for preserving lean mass during weight loss, with studies demonstrating a 50–95% reduction in lean mass loss when combined with caloric restriction [105]. Notably, joint guidelines from the American College of Lifestyle Medicine, American Society for Nutrition, Obesity Medicine Association, and The Obesity Society emphasize that increased protein intake alone is insufficient without structured resistance training, recommending 60–90 min per week of resistance exercise alongside 150 min of moderate aerobic activity [35]. For older adults with sarcopenic obesity, combined exercise and nutritional interventions produce optimal results, improving body fat percentage, appendicular skeletal muscle mass, grip strength, and gait speed beyond either intervention alone [106].

## 6 Redefining therapeutic success in obesity

As pharmacotherapy achieves unprecedented weight reductions, the field must reconsider what constitutes optimal therapeutic success. Weight loss of at least 5% improves metabolic parameters including blood pressure, glycemia, and lipid profiles, while more than 10% is required for meaningful improvements in hepatic steatosis, obstructive sleep apnea, and CV event reduction [107]. Weight loss beyond 15% is associated with lower all-cause mortality and greater quality of life improvements. However, a specific threshold for excessive weight loss remains undefined. More specifically, clinical indicators of potentially excessive weight reduction may include weight loss >5% per month, BMI <18.5 kg/m^2^, caloric intake <800 kcal/day, or evidence of functional impairment, particularly in older adults at risk for sarcopenia [108]. As pharmacotherapy increasingly enables high-magnitude weight loss, the possibility of overtreatment and unintended consequences warrants careful consideration. Very rapid or profound reductions of weight may increase the risk of sarcopenia and functional decline in vulnerable populations, particularly older adults, if resistance training (≥2 days/week) and adequate protein intake (≥1.3 g/kg/day) are not prioritized, and may adversely affect bone health [[109], [110], [111]]. From a metabolic standpoint, substantial weight loss may deepen adaptive thermogenesis and neuroendocrine counter-regulation, potentially increasing susceptibility to regain if treatment is interrupted [38,112]. These considerations reinforce that therapeutic success should be assessed not solely by weight reduction magnitude, but also by preservation of lean mass, functional capacity, nutritional adequacy, and long-term durability. Key domains, limitations of traditional endpoints, and proposed next-generation metrics for evaluating obesity pharmacotherapy are summarized in Table 2.

**Table 2:** Redefining therapeutic success in obesity pharmacotherapy.

Outcome parameter	Traditional measures	Limitations	Next-generation measures	Clinical implications
Weight loss	% total body weight loss	Does not reflect body composition or durability	Rate of loss, stability during the maintenance phase	Avoid excessive or rapid loss; prevent malnutrition
Body composition	BMI, total fat mass	Ignores lean mass loss	Fat-to-lean mass ratio; appendicular muscle mass	Guides need for anabolic support and resistance training
Metabolic health	HbA1c, lipids, BP	May improve transiently	Cardiometabolic risk trajectory; MASLD resolution	Aligns drug choice with comorbidities
Functional status	Rarely assessed	Overlooks frailty and sarcopenia	Strength, gait speed, physical performance	Critical in older adults
Durability	Short-term trial outcomes	Poor predictor of long-term success	Maintenance beyond 1–2 years	Supports chronic treatment models
Safety	GI adverse events	Insufficient to assess long-term safety	CV outcomes, neuropsychiatric effects	Determines population-level adoption
Mode of administration	Injectable vs oral	Oversimplification of adherence and access	Treatment persistence, convenience, scalability, patient preference	Influences adherence, primary-care uptake, and global accessibility
Patient-centered outcomes	Weight satisfaction	Subjective and variable	Quality of life, adherence, treatment burden	Improves shared decision-making
Equity & access	Trial eligibility	Often not representative of real-world populations	Real-world uptake and affordability	Determines population-level impact

**List of abbreviations:** BMI, body mass index; BP, blood pressure; CV, cardiovascular; GI, gastrointestinal; HbA1c, glycated hemoglobin, MASLD, metabolic-associated steatotic liver disease.

Physiological adaptations to weight loss create a persistent “energy gap” that promotes weight regain. In particular, weight reduction triggers disproportionate decreases in resting metabolic rate (approximately 15% beyond what body composition changes would predict), increased ghrelin and decreased anorexigenic hormones, and enhanced metabolic efficiency, i.e. adaptations that may persist as long as the reduced body weight is maintained [112,113]. The weight-loss and maintenance phases represent distinct physiological states requiring different interventions. While weight loss typically occurs within the first 6 months, the subsequent plateau and maintenance phase demands strategies to counteract these homeostatic pressures [114].

The discontinuation of obesity pharmacotherapy consistently results in substantial weight regain, with two-thirds of lost weight typically returning within one year, accompanied by reversal of cardiometabolic improvements [115,116]. The SURMOUNT-4 trial demonstrated that participants switching from tirzepatide to placebo regained 14% of body weight over 52 weeks, while those continuing treatment lost an additional 5.5% [116]. Therefore, current guidelines recommend treating obesity pharmacologically as a chronic disease, analogous to hypertension or dyslipidemia, where medications are not discontinued upon reaching target weight [117]. For patients who must discontinue therapy, strategies including high physical activity (≥60 min/day), self-monitoring, and nutrient-dense dietary patterns may help mitigate weight regain; however, these approaches have not yet been validated in the post–GLP-1 therapeutic setting. Future approaches may include dose reduction for maintenance, intermittent therapy protocols, or sequencing between agents with different mechanisms to optimize long-term outcomes [117].

## 7 Toward personalized obesity pharmacotherapy

As the obesity pharmacotherapy armamentarium expands, matching drug mechanisms to individual patient phenotypes becomes increasingly feasible. Emerging models conceptualize obesity through behavioral and metabolic phenotypes, such as “hungry brain” (impaired satiation), “hungry gut” (impaired satiety), “emotional hunger” (reward-driven eating), and “slow burn” (reduced energy expenditure), offering a rationale for targeted interventions [118]. Although phenotype-based frameworks remain evolving, several practical principles may guide clinical decision-making. Patients with predominant satiety impairment (“hungry gut”) may respond particularly well to GLP-1RAs, including emerging oral formulations. Individuals with impaired satiation or strong central appetite drive (“hungry brain”) may benefit from higher-efficacy multi-agonists or amylin-based combinations that enhance central satiety signaling. In those with reward-driven or emotional eating patterns, centrally acting agents such as naltrexone–bupropion may be considered when appropriate [119,120]. Patients with prominent metabolic comorbidities such as MASLD may derive additional benefit from dual GLP-1/glucagon or triple agonist approaches [[119], [120], [121]]. In older adults or those at risk of sarcopenia, prioritizing resistance training and protein adequacy alongside pharmacotherapy is essential, and future lean-mass–preserving strategies may further refine treatment selection. Although these classifications remain heuristic and require validation through phenotype-stratified trials, they illustrate how emerging pharmacologic diversity may support increasingly individualized care [122].

Setmelanotide exemplifies precision obesity pharmacotherapy already in clinical practice. This melanocortin-4 receptor (MC4R) agonist is FDA-approved for patients aged ≥6 years with obesity due to proopiomelanocortin (*POMC)*, proprotein convertase subtilisin/kexin type 1 (*PCSK1)*, or leptin receptor deficiency, as well as Bardet-Biedl syndrome [118]. By restoring MC4R signaling downstream of genetic defects in the leptin-melanocortin pathway, setmelanotide achieves mean weight reductions of 25.6% in *POMC/PCSK1* deficiency and significant hunger reduction in patients who are typically refractory to conventional therapies [55]. Emerging evidence also supports efficacy in acquired hypothalamic obesity, where structural damage disrupts endogenous α-melanocyte-stimulating hormone production [123]. Unlike incretin-based therapies that target gut-brain hormonal pathways, setmelanotide directly addresses the underlying neuroendocrine defect, illustrating how genotype-directed treatment selection can achieve outcomes unattainable with conventional approaches.

With respect to medical history, cardiometabolic, hepatic, and musculoskeletal comorbidities should guide medication selection. The 2025 American Association of Clinical Endocrinology consensus statement introduces a hierarchy of preferred pharmacotherapy based on obesity-related complications [118]. For example, individuals with T2DM, chronic kidney disease (CKD), or atherosclerotic CV disease should be prioritized for tirzepatide or semaglutide given their demonstrated cardioprotection and renal benefits [119]. Additional therapeutic synergies may also be leveraged, such as topiramate for migraines, naltrexone for alcohol use disorder, and bupropion for smoking cessation, allowing clinicians to address multiple conditions simultaneously [[118], [119], [120]].

Cost, access, and real-world implementation remain critical barriers to translating pharmacologic advances into population-level impact. Second-generation obesity medications are highly expensive, and access is constrained not only by insurance status but also by substantial variability in reimbursement policies across healthcare systems. In many countries, incretin-based therapies are not reimbursed for obesity at all, or coverage is restricted to specific comorbidities (e.g. established cardiovascular disease or T2DM), and may depend on insurance tier, prior authorization requirements or national prescribing criteria. As a result, access to effective obesity pharmacotherapy remains highly heterogeneous across international settings [59,118,124]. First-generation medications (e.g. orlistat, naltrexone–bupropion, and phentermine ± topiramate where approved or available) remain valuable alternatives for patients with stage 1–2 adiposity-based chronic disease (ABCD), particularly when cost or access limit the use of second-generation therapies [118]. For patients with stage 3 ABCD, reflecting more serious obesity-related complications and diseases, second-generation agents, such as semaglutide and tirzepatide, should be used whenever possible if needed for sufficient weight loss to treat these complications. First-generation medications may also serve as maintenance therapy following initial treatment with second-generation agents, offering a more accessible option for long-term weight management [118,119]. Finally, oral formulations and generic availability will be essential for expanding global access, as estimated minimum manufacturing costs for some agents are a fraction of current retail prices [122].

## 8 Controversies, challenges and future perspectives

The rapid evolution of obesity pharmacotherapy has brought to the forefront a series of unresolved controversies that are likely to shape the future trajectory of the field. A central tension lies between the expanding medicalization of obesity and approaches that prioritize its upstream determinants. While pharmacologic therapies now offer unprecedented efficacy at the individual level, critics caution that an increasing reliance on medication risks reframing obesity as a narrowly clinical problem, potentially diverting attention from powerful socio-environmental drivers such as food systems, physical inactivity, and socioeconomic inequities [125]. Accordingly, debate persists over whether pharmacotherapy should function primarily as a first-line intervention or as an adjunct to broader population-level prevention and policy strategies.

Concerns regarding equity and access further complicate this landscape. Despite clear clinical indications, many patients encounter substantial barriers to treatment because of high costs and intermittent drug shortages, whereas individuals with greater financial resources may obtain these agents for non-medical or cosmetic purposes [126]. In parallel, the proliferation of unregulated compounded formulations and celebrity-endorsed supplements has introduced additional safety risks and amplified misinformation, further blurring therapeutic boundaries. Within this context, clinicians are increasingly challenged to balance evidence-based prescribing with ethical stewardship of limited healthcare resources.

Long-term safety considerations represent another critical area of uncertainty. Although currently approved and emerging agents demonstrate acceptable short-term safety profiles, robust long-term data, particularly for multi-receptor agonists, remain sparse. Questions persist regarding potential thyroid, pancreatic, neuropsychiatric, and other off-target effects with prolonged exposure [127,128]. Moreover, it remains unclear whether next-generation agents will confer CV and mortality benefits comparable to those demonstrated with semaglutide in the SELECT trial, or whether therapeutic efficacy will outpace the accumulation of definitive safety evidence [127].

Looking ahead, regulatory and clinical frameworks will need to adapt to an increasingly complex therapeutic landscape characterized by combination regimens, personalized treatment strategies, and outcome measures that extend beyond weight loss alone [127]. Integration of pharmacotherapy with digital health tools, structured behavioral interventions, and “Food is Medicine” initiatives may enhance adherence and long-term effectiveness while reinforcing lifestyle modification [129,130]. Ultimately, sustainable progress in obesity management will depend on a careful balance between innovation and equity, ensuring that transformative therapies alleviate, rather than deepen, the health disparities borne disproportionately by populations most affected by obesity.

## 9 Conclusions

The remarkable success of GLP-1RAs has already marked a turning point in the therapeutic armamentarium of obesity and related cardiometabolic and other diseases. Nonetheless, the next epoch of obesity pharmacotherapy will be defined by multi-receptor strategic combinations, and targeted approaches to better sustain energy balance and body composition, primarily preventing lean mass loss. Amylin analogs, triple agonists, oral incretins, glucagon-based therapies, and anabolic-supportive agents are jointly expanding what is possible in clinical obesity management. The focus will gradually shift from achieving weight loss to maintaining metabolic health, personalizing treatment algorithms, and ensuring broadly accessible, lasting care across diverse populations.

Future directions in obesity pharmacotherapy are likely to converge around several emerging priorities. First, advances in biomarker discovery and validation, including genetic, metabolomic, and gut microbiome signatures, hold promise for enabling precision prescribing that extends beyond current phenotype-based classifications [131,132]. Second, rigorously designed head-to-head comparative effectiveness trials of next-generation agents are urgently needed to inform evidence-based sequencing, switching, and combination strategies in clinical practice. Third, pediatric and adolescent obesity remains a critically understudied domain, as most pharmacologic agents lack regulatory approval or long-term safety data in younger populations, despite the rapidly increasing prevalence and early-life consequences of obesity. Fourth, the neuropsychiatric effects of highly potent anorectic therapies, including potential impacts on mood, cognition, reward processing, and substance use behaviors, require focused investigation that goes beyond routine safety surveillance. Finally, real-world implementation will be essential to determine how obesity pharmacotherapy can be effectively integrated into primary care workflows, community health systems, and low-resource settings. Without such efforts, the robust efficacy observed in clinical trials may fail to translate into meaningful population-level benefit. As this therapeutic landscape continues to evolve, bridging the gap between pharmacologic innovation and equitable, sustainable implementation will remain the defining challenge for the field.

## CRediT authorship contribution statement

**Ioannis G. Lempesis:** Writing – review & editing, Writing – original draft, Validation, Methodology, Investigation, Data curation, Conceptualization. **Maria Dalamaga:** Writing – review & editing, Writing – original draft, Supervision, Project administration, Methodology, Investigation, Data curation, Conceptualization.

## Funding

This work did not receive any specific grant from funding agencies in the public, commercial or not-for-profit sectors.

## Declaration of competing interest

The authors declare the following financial interests/personal relationships which may be considered as potential competing interests: Given her role as Co-Editor-in-chief, Prof Maria Dalamaga had no involvement in the peer review of this article and had no access to information regarding its peer review. Full responsibility for the editorial process for this article was delegated to another journal editor. All authors have no known competing financial interests or personal relationships that could have appeared to influence the work reported in this paper.
